# Crossmodal visual-tactile extinction: Modulation by posture implicates biased competition in proprioceptively reconstructed space

**DOI:** 10.1348/174866409X415942

**Published:** 2010-03

**Authors:** Steffan Kennett, Chris Rorden, Masud Husain, Jon Driver

**Affiliations:** 1 Department of Psychology, University of EssexColchester, UK; 2 Department of Communication Sciences and Disorders, University Of South CarolinaColumbia, South Carolina, USA; 3 UCL Institute of Cognitive Neuroscience, University College LondonUK

## Abstract

Extinction is a common consequence of unilateral brain injury: contralesional events can be perceived in isolation, yet are missed when presented concurrently with competing events on the ipsilesional side. This can arise crossmodally, where a contralateral touch is extinguished by an ipsilateral visual event. Recent studies showed that repositioning the hands in visible space, or making visual events more distant, can modulate such crossmodal extinction. Here, in a detailed single-case study, we implemented a novel spatial manipulation when assessing crossmodal extinction. This was designed not only to hold somatosensory inputs and hand/arm-posture constant, but also to hold (retinotopic) visual inputs constant, yet while still changing the spatial relationship of tactile and visual events in the external world. Our right hemisphere patient extinguished left-hand touches due to visual stimulation of the right visual field (RVF) when tested in the usual default posture with eyes/head directed straight ahead. But when her eyes/head were turned to the far left (and any visual events shifted along with this), such that the identical RVF retinal stimulation now fell at the same external location as the left-hand touch, crossmodal extinction was eliminated. Since only proprioceptive postural cues could signal this changed spatial relationship for the critical condition, our results show for the first time that such postural cues alone are sufficient to modulate crossmodal extinction. Identical somatosensory and retinal inputs can lead to severe crossmodal extinction, or none, depending on current posture.

Following unilateral brain injury, some patients exhibit the phenomenon of extinction, whereby an event on the contralesional side of space can be detected when presented alone, yet is often missed if presented concurrently with a competing ipsilesional event (Bender, 1952; Oppenheim, 1885; see Driver, Vuilleumier, & Husain, 1999; Hillis, 2006, for recent reviews). This behaviour can follow a variety of unilateral lesions, but is classically associated with right-parietal damage (Becker & Karnath, 2007; Karnath, Himmelbach, & Kuker, 2003). Extinction can be found within all the major sensory modalities (Aglioti, Smania, Moro, & Peru, 1998; Bellas, Novelly, & Eskenazi, 1989; Bender, 1952; De Renzi, Massimo, & Pattacini, 1984; di Pellegrino & De Renzi, 1995; Vallar, Rusconi, Bignamini, Geminiani, & Perani, 1994) but might also arise crossmodally (e.g. see Bender, 1952, for an early report of this). Notably, numerous recent studies have observed visual-tactile extinction, whereby an ipsilesional visual event can extinguish a tactile event on the contralesional hand (Bueti, Costantini, Forster, & Aglioti, 2004; di Pellegrino, Làdavas, & Farnè, 1997; Farnè, Bonifazi, & Làdavas, 2005; Farnè, Dematte, & Làdavas, 2005; Farnè, Iriki, & Làdavas, 2005; Farnè & Làdavas, 2000; Farnè, Pavani, Meneghello, & Làdavas, 2000; Làdavas, di Pellegrino, Farnè, & Zeloni, 1998, Làdavas & Farnè, 2004; Maravita, Clarke, Husain, & Driver, 2002; Maravita, Husain, Clarke, & Driver, 2001; Maravita, Spence, Clarke, Husain, & Driver, 2000; Mattingley, Driver, Beschin, & Robertson, 1997; Rapp & Hendel, 2003; Sarri, Blankenburg, & Driver, 2006).

Some experiments have now found that such visuo-tactile crossmodal extinction can be affected by *visible* changes in patients' hand posture, or even by sight of a false ‘rubber’ hand at one location or another (di Pellegrino *et al.*, 1997; Farnè *et al.*, 2000; Làdavas *et al.*, 1998; Làdavas, Farnè, Zeloni, & di Pellegrino, 2000). In standard testing, the left hand is typically placed within its own left hemispace, the right hand in right hemispace, and right visual events near to the right hand are then found to extinguish left tactile events strongly. Di Pellegrino *et al.* (1997) first reported that extinction of the same left touch was reduced if the patient held their own right hand behind their back (and thus out of sight) so that the right visual event no longer appeared close to it. If the right hand remains in place, but the visual event is moved more distant from it (beyond ‘peri-personal’ space, i.e. well beyond the patient's reach) this can also reduce crossmodal visual-tactile extinction (e.g. Làdavas *et al.*, 1998). Such findings have been related to putative ‘hand-centred’ multi-sensory representations of space, possibly like those found by single-cell recordings within the monkey-brain (e.g. Graziano & Gross, 1993).

Notably, in all of the studies showing modulation of crossmodal visual-tactile extinction to date, by spatial manipulations of how the hands are located relative to visual events, the effective changes in hand posture (or apparent hand posture, see below) have been *visible*. Indeed, one study reported that when the hands were occluded, comparable manipulations of hand position no longer modulated crossmodal extinction (Làdavas *et al.*, 2000). This, together with the findings when manipulating the location of false rubber hands (Farnè *et al.*, 2000) might be taken to imply an essential role for vision in reconstructing the location, or apparent location, of multi-sensory events (both visual and tactile) that can contribute to crossmodal extinction. However, a critical role for vision alone in reconstructing spatial representations would appear to contrast with some recent evidence on crossmodal attentional competition in healthy subjects, which shows that postural manipulations can change visual-tactile interactions even when those postural changes are unseen (e.g. Kennett, Spence, & Driver, 2002; Macaluso, Frith, & Driver, 2002) and are thus coded proprioceptively instead.

Here we addressed such issues by introducing a novel spatial manipulation to the study of crossmodal visual-tactile extinction, while using computer-controlled visual and tactile stimuli. Unlike prior studies that had altered the location of either or both hands in external space (e.g. di Pellegrino *et al.*, 1997; Farnè *et al.*, 2000; Làdavas *et al.*, 1998, 2000), we held those hand location aspects constant. Moreover, unlike prior studies that had changed the retinotopic visual locations of any visual events (e.g. Mattingley *et al.*, 1997), we also held those retinotopic visual aspects constant. How then, did we vary the relative location of tactile and visual events, without changing neither somatotopic nor retinotopic inputs? In fact this was achieved very simply (see Figure 1), by varying the direction of the patient's eyes/head. In the ‘deviated’ posture (see Figure 1a), the patient's eyes and head were deviated towards the left. Crucially, we arranged that the possible experimental visual events (a fixation light, plus possible flickering of another light in the visual periphery) were shifted along with gaze, so that they appeared at exactly the same *retinal* location as before (for the peripheral events, always at 20° of retinal eccentricity on the horizontal visual meridian). But in the new deviated posture (Figure 1b), the possible right visual field (RVF) visual event now fell at the same external location as the left-hand touch (which was a computer-controlled touch that could not be seen, see below). Hence if crossmodal extinction in part reflects pathologically biased competition between different *locations in the external world*, such competition might now be reduced or eliminated (leading to less crossmodal extinction), as the peripheral visual and tactile events actually now share the same external location rather than different, competing external locations. By contrast, if a RVF event at 20° of retinal eccentricity on the horizontal meridian will always compete with the left-hand touch (when hand posture itself is unchanged), then no change in crossmodal extinction should be observed. For instance, the left-hand touch and the RVF visual event would still project to opposite hemispheres in the deviated posture (as the somatotopic and retinotopic locations of the tactile and visual event are in themselves unchanged). Such hemispheric-projection factors have often been suggested to be a key aspect underlying extinction after unilateral brain injury (e.g. see Kinsbourne, 1987; Marzi *et al.*, 1997).

**Figure 1:**
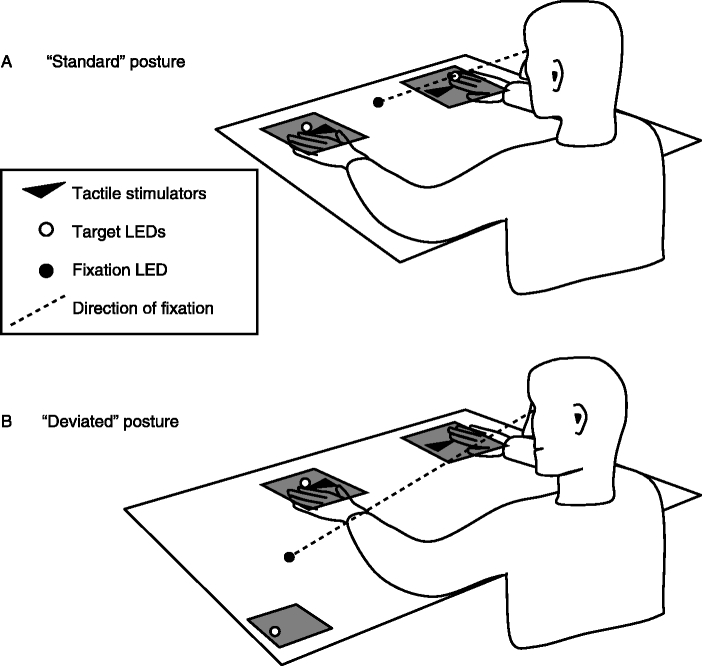
Schematic diagram showing the layouts used in Expt 1. Shaded squares (shown transparent here, but opaque in the experiment) depict the small shields that occluded the tactile stimulators and stimulated fingers from the patient's view in Expt 1. Note that the right-visual-field visual stimulus becomes positioned over the left hand in the deviated posture, but that the retinotopic visual location and somatotopic tactile location of the experimental visual and tactile stimuli are themselves unchanged across postures. In Expt 2 the whole array, plus the hands, arms, and shoulders of patient JM, were occluded by a large sheet. The leftmost shaded square represents a dummy shield on which was placed the left visual-field stimulus for the deviated posture.

Here we applied computer-controlled visual and tactile stimulation in a detailed single-case study, to test the outcome of the new spatial manipulation. In Expt 1, the hands of our patient were occluded (to prevent vision of any tactile events). In Expt 2, we occluded not only her hands but the entire scene (other than the fixation light, and the possible flashing of the peripheral lights) by means of a large black cloth. This meant that, particularly in the critical conditions of Expt 2, the only information about the realignment of the patient's left hand with the RVF light that could be available to the patient was via proprioceptive postural cues. Hence if our novel spatial manipulation were found to modulate crossmodal visual-tactile extinction substantially, this would demonstrate that purely proprioceptive factors can contribute to the spatial representations in which crossmodal extinction arises. As emphasized by our review of the recent crossmodal-extinction literature above, this purely proprioceptive nature of the critical manipulation contrasts with previous studies of crossmodal visual-tactile extinction, which have all typically made substantial visual changes (e.g. manipulating whether the hands, or alternatively rubber-hands, are visible and where they are seen) when studying modulations of crossmodal extinction; leading to some suggestions that proprioception might make little or no contribution on its own (e.g. Làdavas *et al.*, 2000).

Finally, in addition to testing for crossmodal extinction of left-hand touch by a visual event in the retinal RVF, we also assessed unimodal tactile extinction (i.e. between concurrent touches on both left and right hand, without any competing visual event), in order to determine whether the spatial manipulation shown in Figure 1b might have a highly specific effect on crossmodal extinction only, as we anticipated (due to the spatial realignment in the external word of the left-hand touches with the RVF visual event, see Figure 1b); or whether instead turning leftwards, as in the deviated posture, merely produces some less specific advantage that applies to left-hand touch more generally (see also Larmande & Cambier, 1981; Vaishnavi, Calhoun, 2001). For completeness, we also assessed unimodal visual extinction in both postures also (see Figure 1), and any extinction from right-hand touch upon left visual field (LVF) visual events; none of the latter was found.

## Experimental procedures

### Case report

**Figure 2:**
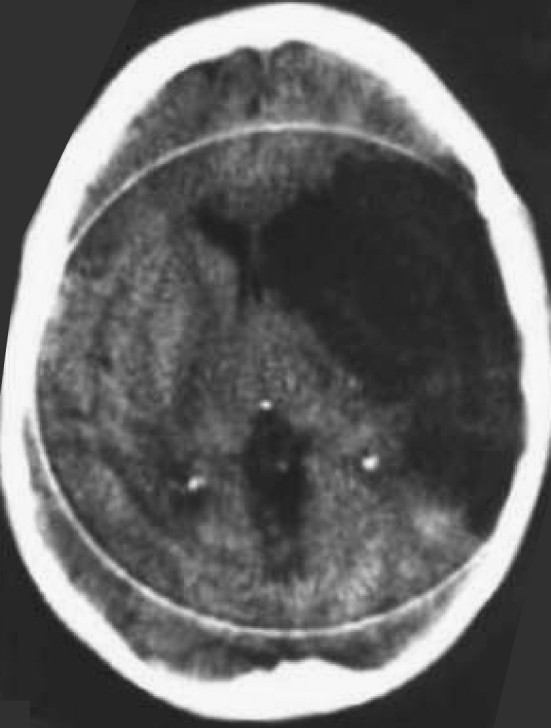
Transverse section from the cranial CT scan of JM. The darker area is consistent with infarction in the territory of the right middle cerebral artery. The lesion involves regions of the parietal, temporal, and frontal lobes (note that the bright outline circle is an artifact of the image production).

Patient JM was a 62-year-old right-handed woman, selected for this study due to her highly consistent crossmodal extinction of left-hand touch by right visual events, persisting in the chronic period long after her right hemisphere stroke. Six years prior to testing she had presented with a sudden-onset left hemiparesis, consistent with right hemisphere stroke, which affected her arm more than her leg. Visual fields were noted to be full on manual confrontation testing. A computed tomography (CT) scan demonstrated an extensive low attenuation region consistent with infarction in the territory of the right middle cerebral artery (see Figure 2). The lesion compromised parts of the right parietal, temporal, and frontal lobes.

On clinical examination at the time of the current study, she had a dense left-sided pyramidal weakness. Visual fields were full, but she demonstrated highly reliable extinction of left-sided stimuli on simultaneous bilateral visual confrontation. She was able to detect light tactile stimulation of her left hand reliably. However, she demonstrated complete left-sided tactile extinction clinically when both hands were concurrently touched lightly by the examiner. Pilot testing using the same computerized visual and tactile stimuli as employed in the current investigation (see below) also found that JM showed clear crossmodal extinction of left tactile events by RVF visual events, along with unimodal extinction within both vision and touch when using computerized stimuli also, as per below.

At the time of testing, there was no evidence of visual neglect on drawing, line bisection, Behavioural Inattention Test (BIT) star cancellation or Mesulam shape cancellation.

### Stimuli and apparatus

Figure 1 depicts the experimental layout schematically. The patient sat in a quiet room at a table. Her arms were placed in a comfortable, symmetrical posture on a tabletop, with hands placed palm-down 30 cm apart (each at 20° eccentricity when fixating centrally). Tactile stimulation was provided by unseen (occluded, see below) metal rods each propelled by a 12 V solenoid to deliver brief punctate strikes to the skin (2 mm^2^ contact area). One rod was positioned next to each hand so it could firmly strike the surface of the middle segment of the index finger, which was velcro-strapped in place. Small occluding shields covered the tactile devices and stimulated hands (see Figure 1), precluding view of any small movement associated with tactile stimulation. Possible peripheral visual stimuli were generated via two green light emitting diodes (LEDs), each 5 mm in diameter, that provided brief computer-controlled visual events. These visual stimuli were arranged in one of two possible ways (see ‘standard’ or ‘deviated’ layouts in Figure 1). The peripheral LEDs were mounted at 20° on either side of fixation, along the horizontal meridian of the visual field. A third LED (orange) was positioned to define fixation. In the ‘deviated’ posture, the entire visual array was shifted 40° to the left and JM's eyes (and head) were rotated to attain the new fixation position, with the two green LEDs shifted along with the orange fixation LED, to ensure identical retinal positions for the eccentric visual LEDs relative to the new fixation point. The possible tactile stimulation was unchanged across the two postures, both somatotopically and also in external space. The deviated posture was deliberately designed such that the RVF visual stimulus now shared the same location as the left tactile stimulus in external space (see Figure 1b), despite having an unchanged *retinal* position (still 20° eccentricity in the RVF, along the horizontal meridian of the visual field) due to the shift in fixation with the turned eyes/head.

Pilot testing identified suitable event durations for producing reliable within-modality extinction in our patient. All tactile events were powered for 70 ms and all visual events were 80 ms in duration throughout the main two experiments.

Expt 2 was similar to Expt 1, but had two additional conditions (again involving the ‘standard’ and ‘deviated’ postures, see Figure 1) in both of which a large black occluding sheet was now attached to JM's shoulders, stretching out horizontally in all directions, including forwards above and well beyond the hands. Due to this occluding sheet, the three LEDs were now the only potentially visible part of the apparatus, with all other apparatus (and also the shoulders, arms, and hands) now entirely occluded below the large black sheet. Hence no information was available to the patient about the relative position of her hands with respect to the possible visual events, in these occluding-sheet conditions, other than from proprioception (see Introduction for why this was important).

### Procedure

Patient JM was instructed to fixate the orange light during all trials. Successful fixation was monitored and confirmed by the experimenter, who sat opposite JM and ensured that appropriate fixation was achieved before initiating each trial of brief stimulation. (Any peripheral visual stimuli were 80 ms in duration and any tactile stimuli lasted 70 ms, so stimulation in either modality was too brief to allow any saccades prior to the stimulation finishing. But careful observation by the experimenter confirmed the patient's success in maintaining fixation in any case, see below). Continuous white noise at 70 dB(A) completely masked the slight sounds made by operation of the tactile devices, so that tactile events could only be detected by touch.

On each trial, either one side (unilateral), both sides (bilateral) or neither side (‘catch’ trials) could be stimulated unpredictably. Unilateral trials were randomly *either* tactile or visual (i.e. no more than one modality was ever stimulated on a given side) and could occur unpredictably on the left or right side. Bilateral trials were of four types: unimodal visual, unimodal tactile, left visual/right tactile, or left tactile/right visual. Thus any one of nine trial types could be presented unpredictably (four unilateral possibilities, four bilateral, or catch trials). The participant was required to give an unspeeded response by saying ‘left’, ‘right’, ‘both’, or ‘none’, according to her conscious detection of stimulus events, but regardless of their modality. Within a block, each trial type was presented 8 times, with the exception that the two bimodal-bilateral trial types (critical for assessing any crossmodal extinction) were presented 12 times each. Each block thus contained 80 trials, presented in random order.

JM was familiar with the ‘standard’ posture used in this experiment, from previous pilot testing. She was introduced, prior to Expt 1, to the ‘deviated’ posture with two short practice blocks (36 trials each) of unimodal visual trials. A further short practice block was then presented with the same design as the experimental blocks (i.e. both unimodal and bimodal trials intermingled), except that JM was required to informally describe her experience after each trial rather than give the formal responses. The purpose of this practice was to familiarize JM with the left and right verbal labels assigned to each stimulus, which were defined according to their anatomical inputs (e.g. unilateral left hand tactile stimuli or unilateral LVF visual stimuli were both called ‘left’ in both postures). Following this practice session, JM was presented with six experimental blocks: three blocks in the deviated posture followed by three blocks in the standard posture. This fixed order was controlled for in the subsequent Expt 2. The total testing time was 1 h.

No additional practice was required prior to Expt 2, which started 3 weeks after Expt 1. Expt 2 was conducted over two sessions separated by a week. In addition to repeating the ‘standard’ (1) and ‘deviated’ (2) conditions from Expt 1, these two conditions were also implemented again but now with the arms and other aspects of the visual scene completely occluded by the large black sheet (thus providing two occluded conditions 3 and 4, one for each postural arrangement). JM underwent one block (of 80 trials each, made up of the same trial types as in Expt 1) for each of the four arrangements in the first session; and another block of each condition in the second session (overall order: 3–4–1–2, 1–2–3–4). The total testing time was 1 h in each session.

## Results

Data from both experiments were subjected to the same analyses. Rare trials (0.8%) with unsatisfactory fixation (the patient shifting gaze just as the experimenter initiated a trial) were excluded. Performance was near ceiling for all unilateral trials, confirming that neither touch nor vision were so impaired in JM as to prevent sensation of the computer-controlled stimuli on either the contra- or ipsilesional side. JM rarely made false positives for stimuli not presented. On catch trials JM correctly responded ‘none’ for all such trials throughout both experiments; on unilateral trials, she responded ‘both’ only twice for 192 trials in Expt 1 and only six times for 256 trials in Expt 2.

As is conventional in studies of unilateral extinction, responses were re-coded into ‘detected’ or ‘not detected’ for particular stimulus types (for instance, for left stimuli, ‘left’ or ‘both’ responses would both count as including left detection, while ‘none’ or ‘right’ would both count as the left stimulus not being detected). Extinction was tested for by comparing performance in bilateral trials with that in unilateral trials, assessing whether contralesional (i.e. left-sided) stimuli were detected less often for bilateral than unilateral trials. The bilateral trials of most interest for our hypotheses are those with a tactile event on the left hand and a concurrent visual event in the RVF. In the ‘standard’ posture these two events took place in distinct locations in external space. However, in the ‘deviated’ posture these same two events (identical in terms of the anatomical inputs they stimulated to those in the standard posture, i.e. 20° into the RVF on the horizontal retinal meridian for the visual event, plus punctate touch on the left-hand for the tactile event) now fell close to each other in external space. Table 1 provides the data for left touch/right visual trials in both postures, together with the corresponding unilateral left tactile trials, from Expt 1. Table 2 shows the same datasets for Expt 2, now subdivided according to whether the arms were covered by the occluding-sheet or not. The analogous datasets from unimodal, tactile bilateral trials are also shown. The raw responses given in all conditions are displayed in Tables A1 and A2 (see Appendix).

**Table 1:** Number (excluding eye-movement trials) of left and right event detections in both standard and deviated postures from left tactile/right visual and left tactile/right tactile bilateral trials in Expt 1, with the corresponding unilateral performances

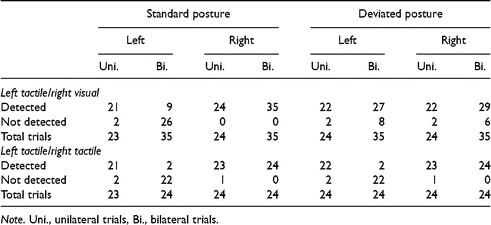

**Table 2:** Number (excluding eye-movement trials) of left and right event detections with arms covered and uncovered, in both standard and deviated postures from bilateral trials in Expt 2, with the corresponding unilateral performances

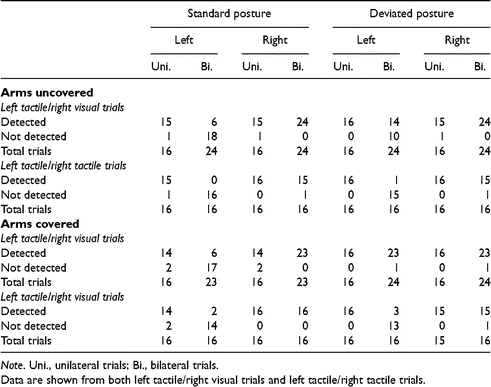

**Figure 3:**
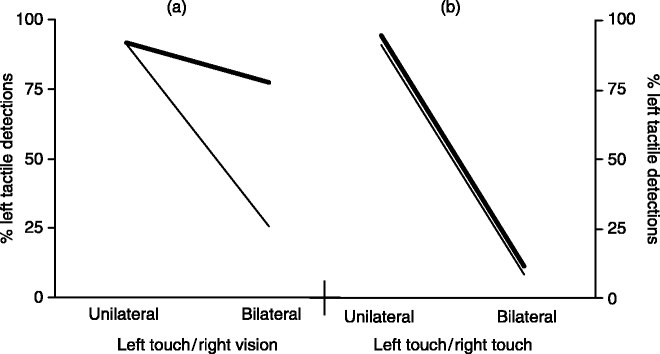
Percentage left tactile detections in unilateral and bilateral trials for Expt 1. Separate plots are presented for (a) left touch/right vision crossmodal trials and for (b) left touch/right touch unimodal trials. Bold lines depict performance in the deviated posture, thinner lines are for the standard posture. Note that the actual data for left touch/right touch (in b) overlap perfectly; the slight vertical offset between the two lines is added here only so that both can be seen.

**Figure 4:**
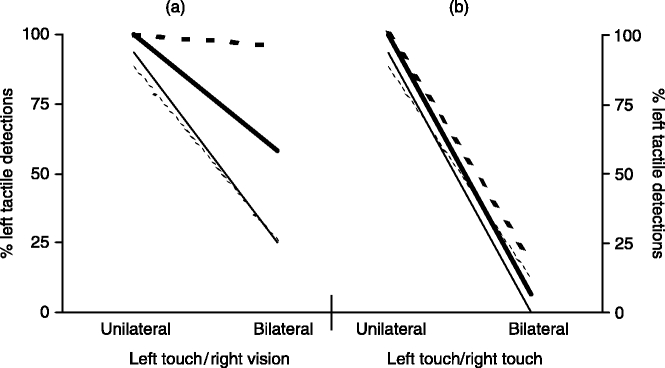
Percentage left tactile detections in unilateral and bilateral trials for Expt 2. Separate plots are presented for left touch/right vision crossmodal trials (a) and left touch/right touch unimodal trials (b) Bold lines depict performance in the deviated posture, thinner lines are for the standard posture. Dotted lines (thick for deviated posture, thin for standard) are for when the arms and hands were completely occluded with a sheet.

The critical results are graphed as percentages in Figures 3 and 4. Figure 3a shows that in Expt 1, crossmodal extinction of left touch by RVF visual events (i.e. the large drop of left-touch detection in the presence of a right visual competitor, as compared to a left touch presented alone unilaterally) was substantial in the standard posture, but was strikingly reduced in the deviated posture. By contrast, Figure 3b shows that our novel postural manipulation had absolutely no impact on unimodal, within-modality tactile extinction of left-hand touch by a concurrent right touch (in fact the data from the two conditions overlap perfectly; the slight offset between the lines of Figure 3b is added to allow both to be seen clearly). Such within-modality, purely tactile extinction remained severe for both postures.

The results for Expt 2 (see Figure 4) essentially replicated the data from Expt 1, while further showing that the reduction in crossmodal extinction of left-hand touch by a RVF event due to the novel deviated posture could be obtained even in the sheet occluded condition (see, in particular, the solid dashed line in Figure 4a). Once again, the postural manipulation had no effect on unimodal, within-modality tactile extinction (Figure 4b), while the occluding-sheet manipulation did not alter performance. Thus the postural manipulation only affected *crossmodal* extinction of left-hand touch by the RVF visual event (compare Figures 3a and 4b with 3b and 4b, respectively), and this remained true even with the occluding sheet.

These conclusions were confirmed by chi-squared tests. Highly reliable unimodal tactile extinction was observed for left tactile/right tactile bilateral trials within both experiments, regardless of posture and also of whether the arms were occluded from view with the sheet or not (all tests showed χ^2^(1)>17.9, *p* <.001). In contrast, crossmodal extinction for left tactile/right visual trials depended critically on posture. In Expt 1, crossmodal extinction was reliable only for the standard posture [standard: χ^2^(1, *N* = 58) = 23.9, *p* <.001; deviated: χ^2^(1, *N* = 59) = 2.1, *p* = .14]. In Expt 2, in the critical new situation when the arms were completely occluded by the sheet, crossmodal extinction for left tactile/right visual trials again depended on the posture, only being significant in the standard posture, [χ^2^(1, *N* = 39) = 14.2, *p* <.001], not the deviated χ^2^(1, *N* = 40) = 0.7, *p* = .4].

For completeness, we also implemented a signal detection theory analysis (see also Olson, Stark, & Chatterjee, 2003; Ricci & Chatterjee, 2004; Ricci, Genero, Colombatti, Zampieri, & Chatterjee, 2005; Sarri *et al.*, 2006) on our most critical effect, namely crossmodal extinction of left-hand touch by a RVF visual event that depended on posture. Briefly, by calculating the hit rate (proportion of left tactile events successfully detected) and the false alarm rate (proportion of no-left-event trials in which a left stimulus is erroneously reported), it is possible in principle to determine whether performance differences across conditions can be attributed to genuine changes in sensitivity for the stimulus (corresponding to the *d* ′ *parameter*) rather than a bias to give one response more than another (Green & Swets, 1966). For cases in which false-alarm rates were zero, we followed the conservative convention (as recommended by Macmillan & Creelman, 1991; Snodgrass & Corwin, 1988) of added a count of 0.5 to all cells within a single analysis. This same approach was recently applied successfully to crossmodal extinction data, for the first time, by Sarri *et al.* (2006). Applying it here revealed that sensitivity (*d* ′) to left-hand touch in the crossmodal-extinction situation did depend on posture: a significant reduction in *d* ′ for left-hand touch when a RVF visual event was added was found only for the standard posture (standard: unilateral *d* ′ = 3.31; bilateral *d* ′ = 0.92; extinction effect on *d* ′ with *p* = .003) with this reduction in tactile sensitivity due to crossmodal extinction from a RVF visual event being eliminated in our novel deviated posture (unilateral *d* ′ = 3.34; bilateral *d* ′ = 2.00, difference in *d* ′ no longer significant).

Note that the chi-square and *d* ′ analysis reported above only reveal our hypothesized result in terms of a null-effect. Therefore, we conducted a final analysis where the hypothesized effect could be detected as a statistically significant interaction effect. Specifically, our *a priori* prediction was that *crossmodal* extinction of left-hand touch by a RVF visual event would be attenuated when the posture was deviated. To examine this, we performed logistic regression analyses on the left tactile detection data from bilateral trials for each experiment. This statistical method allows data involving a dichotomous dependent variable (e.g. detected vs. not detected) to be analysed in a multi-factorial way (analogous to analysis of variance). For each experiment a series of hierarchical models were compared. The best fitting model was determined by following the stepwise procedure advocated by Hosmer and Lemeshow (1989). For Expt 1 the analysis has factors of right event (touch vs. vision) and posture (standard vs. deviated). The resultant best fit is the saturated model, containing terms for the two main effects and the interaction between them. Only the term for the interaction between posture and right event modality approached significance [χ^2^(−2 log LR; *df* = 1) = 3.8; *p* = .050]. Neither of the main effects modelled the data well (both χ^2^<1.1; both *p*s>.2). The interaction term thus best describes the pattern of the data, confirming that extinction for the left tactile event was significantly reduced in the deviated posture *only when the right event was visual*; that is, only in the crossmodal situation, not for unimodal tactile extinction.

The data from Expt 2 were similarly analysed with factors of right event (touch vs. vision), posture (standard vs. deviated), *plus* occlusion (occluded sheet vs. no occlusion). The resultant best fitting model has the three main effects and two two-way interactions. Within this model none of the main effects contribute significantly (all χ^2^<0.9; all *p* >.3). The only significant term in the model was the interaction between posture and right event modality [χ^2^(−2 log LR; 1) = 4.0; *p* = .04], confirming once again that the effect of posture on extinction again applied only when the right event was visual (the crossmodal case) and not when it was tactile (the unimodal case). The occluding sheet did not change this pattern (no main effect or interaction involving that factor). These logistic-regression analyses further confirm the conclusions reached earlier above, when using either the conventional approach of a chi-square test for each unilateral/bilateral 2 × 2 contingency table, or considering changes in sensitivity as measured by *d* ′.

## Discussion

We tested in detail a right hemisphere patient who showed chronic and reliable crossmodal extinction of left tactile events by right visual events (see also Bender, 1952; Bueti *et al.*, 2004; di Pellegrino *et al.*, 1997; Farnè, Bonifazi *et al.*, 2005; Farnè, Dematte *et al.*, 2005; Farnè, Iriki *et al.*, 2005; Farnè & Làdavas, 2000; Farnè *et al.*, 2000; Làdavas & Farnè, 2004; Làdavas *et al.*, 1998; Mattingley *et al.*, 1997; Rapp & Hendel, 2003) as confirmed here with computer controlled stimuli (see also Maravita, Clarke *et al.*, 2002; Maravita *et al.*, 2001; Maravita *et al.*, 2000; Sarri *et al.*, 2006). Our critical new finding was that such crossmodal extinction was very strongly modulated by a novel postural manipulation, that changed the relative spatial positions of the tactile and visual events in external space, but without changing the somatotopic nature of the tactile events (on the left hand) nor the retinal nature of the visual events (in the RVF), and thus without changing which hemisphere they would project to.

In the novel deviated posture, fixation was shifted leftwards (with the patient turning her head and eyes correspondingly), and any visual events were shifted along with fixation, so that their *retinal* locus remained unchanged (see Figure 1). As a result, the peripheral visual event in the RVF now fell as the same external location as the touched left hand. Crossmodal extinction was dramatically reduced in this situation (see Figures 3a and 4a), even though unimodal tactile extinction was unchanged by the novel postural manipulation (see Figures 3b and 4b) and unimodal visual extinction was, similarly, not reduced (see Tables A1 and A2). Moreover, the dramatic impact of posture on crossmodal extinction of left-hand touch by a RVF visual event, in particular, was still found even when *all vision of posture* was eliminated, via the occluding sheet (Expt 2). This implies that purely proprioceptive information about current posture can be sufficient to dramatically alter crossmodal extinction, implying that proprioception can contribute to the spatial representations in which crossmodal extinction arises. Since the whole head was turned to the new posture, the position of the eye within the head was constant across conditions. Thus the source of the proprioceptive signal for the deviated posture was likely to be in the neck rather than the eyes.

The fact that our postural manipulation did *not* affect unimodal tactile extinction or improve unimodal visual extinction (as examined with interleaved trials within the same experimental blocks), but ameliorated only *crossmodal* extinction of left-hand touch by RVF visual event, is important for showing the specificity of our current findings. This outcome makes sense when one considers that the deviated posture did not change the relative locations of the two hands in the external world, only the relative location of the RVF visual event and the left-hand touch in the external world. However, one should consider how our findings might relate to other reports (Larmande & Cambier, 1981; Vaishnavi *et al.*, 1999, 2001) that unimodal left tactile extinction can sometimes be reduced by gazing leftwards. Those studies differed from the present postural manipulation in several important methodological respects. First, patients gazed *directly* at the left hand, rather than beyond it as here, which might have boosted attention towards the left hand in the other studies. Second, it is possible that subtle visual cues about tactile stimulation might have inadvertantly been present in some (though not necessarily all) of those other studies, some of which had used typical clinical confrontation methods, whereas any such inadvertant cues were completely eliminated here by the use of computer-controlled stimuli and occlusion. In any case, the most important finding of the present study is unequivocal; in a carefully designed, within-patient, within-experiment comparison of unimodal versus crossmodal extinction, for intermingled trial types, only crossmodal extinction of left-hand touch by a RVF visual event was affected by our novel posture manipulation. Our results thus show that a critical factor in determining crossmodal extinction is the relative location of events in external space, not purely their initial hemispheric projections, (cf. Kinsbourne & Jeannerod, 1987; Marzi *et al.*, 1997), which remained the same across our postural manipulation. Moreover, we replicated our novel finding across two separate experiments, and showed via the occlusion manipulation in Expt 2 that our critical new finding must depend on proprioceptive information about current posture.

It remains possible that the position of the visual and tactile events in external space might have been coded according to allocentric or egocentric coordinates (e.g. trunk-centred coordinates). However, any correct spatial coding can only be derived by combining the changing proprioceptive information with the invariant tactile and visual information. The improved performance in the deviated posture might then result from at least three mechanisms: Firstly, the dominating RVF visual event might enhance the representation of the left hand (as proposed by Làdavas *et al.*, 1998); Secondly, the RVF visual stimulus might be represented less strongly due to its new location to the left of the trunk midline (cf. Karnath, Schenkel, & Fischer, 1991); Lastly, the attentional demands of the task might be reduced when the two stimuli are at a single spatial location, in the deviated posture, as opposed to spread across two locations, in the standard posture.

Our results argue against previous suggestions that extinction might be caused simply by some pathological form of sensory masking reflecting stimulus strength (e.g. Battersby, Bender, Pollack, & Kahn, 1956). Such accounts might argue, for instance, that a relatively weak touch to one hand (or a pathologically weakened tactile input) can be detectable when presented alone, yet go unnoticed if presented together with a much stronger stimulus, due to some form of masking. However, such accounts would presumably predict the same (or even greater) masking for two stimuli when presented closer together in external space, as in the novel posture used here. But our results clearly go against this. The RVF event was actually *closest* to the left-hand tactile event (in external space) in precisely those bimodal conditions where left tactile performance was best. This renders implausible any sensory-masking account for the tactile-visual extinction observed here. By contrast, the improved performance when the potentially competing multimodal events fell at the same external spatial location might accord with several recent neuroscience demonstrations of enhanced responses to multimodal stimulation when arising from a common external location, even across changes in posture (Eimer, Cockburn, Smedley, & Driver, 2001; Macaluso, Driver, van Velzen, & Eimer, 2005; Macaluso, Frith, & Driver, 2000; Stein & Meredith, 1993); and also accord with recent normal behavioural studies of spatial tactile-visual interactions across changes in posture (Driver & Spence, 1998; Kennett, Eimer, Spence, & Driver, 2001; Kennett *et al.*, 2002; Maravita, Spence, Kennett, & Driver, 2002a, b; Spence & Driver, 1998; Spence, Pavani, & Driver, 2000).

The present results are broadly consistent with the influential proposals of di Pellegrino *et al.* (1997) and Làdavas *et al.* (1998) concerning crossmodal extinction, following their manipulations of *arm* posture (rather than of head/gaze posture, as here. See also Farnè *et al.*, 2000; Làdavas *et al.*, 2000). However, one critical difference is that here we found crossmodal extinction to be dramatically modulated by posture *even when the arms were unseen* (Expt 2, occlusion conditions), so that only *proprioception* could signal the current posture, and thus the new spatial relationship of the visual and tactile events. This goes against some suggestions (Làdavas *et al.*, 1998, 2000) that postural effects on crossmodal extinction might not involve proprioception. That suggestion was based on findings that crossmodal extinction was only modulated by changing hand posture when those changes in hand posture could be seen (Làdavas *et al.*, 2000). Such results might be reconciled with the current finding of a proprioceptive influence from head/gaze direction, however. Unlike our hands, which are often visible, we do not usually see our own head and gaze direction (except rarely as reflections in mirrors, etc.), but must typically rely instead on proprioceptive cues concerning this aspect of our posture. That might explain why such proprioceptive cues about head/gaze posture in particular are so important, and can strongly modulate crossmodal extinction, as here. Moreover, some multi-sensory cells in the monkey brain (within premotor cortex) have been observed to show proprioceptive updating of stimulus locations even in darkness (Graziano, Hu, & Gross, 1997) in relation to head/gaze postural changes. Finally, we note that while head and gaze direction might be a relatively novel manipulation for studies of extinction (though see also Larmande & Cambier, 1981; Vaishnavi *et al.*, 1999, 2001), head and gaze movements are in fact very common in daily life, and so must often be compensated for, via proprioceptive cues, if the spatial relationships of visual and tactile events in the world are to be updated and reconstructed (Driver & Spence, 1998; Spence & Driver, 1998).

In conclusion, this single-case study demonstrates unequivocally that the relative location of tactile and visual stimuli in external space can be a particularly important determinant of crossmodal extinction, even when the initial hemispheric projections for the competing stimuli (and also their somatotopic and retinotopic properties, for tactile and visual events, respectively) are held strictly constant. The relative location of tactile and visual stimuli in external space can evidently be reconstructed, across changes in head/gaze posture, prior to the level at which crossmodal extinction arises. Our study shows for the first time that purely proprioceptive cues about current posture can influence visual-tactile extinction, in situations where posture is unseen (as with the occluding sheet in Expt 2). Finally, while the postural change dramatically modulated crossmodal extinction of left-hand touch by RVF visual events, it had no effect whatsoever on unimodal tactile extinction, consistent with the relative location of the tactile events on the two hands in the external world being unchanged by our postural manipulation (unlike the changed relative location of visual relative to tactile events), thus further underlining the importance of external spatial location in extinction, and the crossmodal specificity of our effects.

## Appendix

**Table A1:** Responses given in Expt 1 by condition

**Table A2:** Responses given in Expt 2 by condition
